# Psychosocial impact of breast/ovarian (*BRCA1/2*) cancer-predictive genetic testing in a UK multi-centre clinical cohort

**DOI:** 10.1038/sj.bjc.6602207

**Published:** 2004-10-26

**Authors:** M Watson, C Foster, R Eeles, D Eccles, S Ashley, R Davidson, J Mackay, P J Morrison, P Hopwood, D G R Evans, and Psychosocial Study Collaborators

**Affiliations:** 1 Department of Psychological Medicine, Royal Marsden NHS Foundation Trust, London & Sutton, SM2 5PT, England; 2 Macmillan Research Unit, School of Nursing and Midwifery, University of Southampton, SO17 1BJ, England; 3 Translational Cancer Genetics Team & Cancer Genetics Unit, Royal Marsden NHS Trust, London & Sutton, SM2 5PT, England; 4 Wessex Clinical Genetics Service, Princess Ann Hospital, Southampton, SO16 5YA, England; 5 Department of Computing, Royal Marsden NHS Trust, Sutton, SM2 5PT, England; 6 Institute of Medical Genetics, Yorkhill NHS Trust, Glasgow, G3 8SJ, Scotland; 7 Genetics Centre, Institute of Child Health, London, WC1N, England; 8 Medical Genetics, Belfast City Hospital, Belfast, BT9 7AB, Northern Ireland; 9 Christie Hospital, Manchester, M20 4BX, England; 10 Department of Medical Genetics, St Mary's Hospital, Manchester, M13 0JH, England

**Keywords:** predictive genetic testing, worry, risk management, psychosocial issues, BRCA

## Abstract

This multi-centre UK study assesses the impact of predictive testing for breast and ovarian cancer predisposition genes (BRCA1/2) in the clinical context. In the year following predictive testing, 261 adults (59 male) from nine UK genetics centres participated; 91 gene mutation carriers and 170 noncarriers. Self-report questionnaires were completed at baseline (pre-genetic testing) and 1, 4 and 12 months following the genetic test result. Men were assessed for general mental health (by general health questionnaire (GHQ)) and women for general mental health, cancer-related worry, intrusive and avoidant thoughts, perception of risk and risk management behaviour. Main comparisons were between female carriers and noncarriers on all measures and men and women for general mental health. Female noncarriers benefited psychologically, with significant reductions in cancer-related worry following testing (*P*<0.001). However, younger female carriers (<50 years) showed a rise in cancer-related worry 1 month post-testing (*P*<0.05). This returned to pre-testing baseline levels 12 months later, but worry remained significantly higher than noncarriers throughout (*P*<0.01). There were no significant differences in GHQ scores between males and females (both carriers and noncarriers) at any time point. Female carriers engaged in significantly more risk management strategies than noncarriers in the year following testing (e.g. mammograms; 92% carriers *vs* 30% noncarriers). In the 12 months post-testing, 28% carriers had bilateral risk-reducing mastectomy and 31% oophorectomy. Oophorectomy was confined to older (mean 41 yrs) women who already had children. However, worry about cancer was not assuaged by surgery following genetic testing, and this requires further investigation. In all, 20% of female carriers reported insurance problems. The data show persistent worry in younger female gene carriers and confirm changes in risk management consistent with carrier status. Men were not adversely affected by genetic testing in terms of their general mental health.

Following the identification of two predisposition genes (BRCA1 and BRCA2) that increase the risk of breast and/or ovarian cancer (Miki *et al*, 1994; Wooster *et al*, 1995), there has been a gradual introduction of genetic testing into the clinical context. This has presented a number of challenges for the medical community, patients and their families. Female carriers of a BRCA1/2 genetic mutation, not already affected by cancer, have up to 85 and 60% lifetime risks of developing breast and ovarian cancer, respectively (Ford *et al*, 1998). They are at increased risk of early-onset disease (often pre-menopausal) and bilateral breast cancer (up to 64% by age 70 years) (Ford *et al*, 1994). Female BRCA1 carriers who have had breast cancer before age 60 have an ovarian cancer risk of 44% by age 70 (Ford *et al*, 1994). Male carriers have an increased risk of prostate cancer (6% by age 70) and male and female BRCA1 carriers have an increased risk of bowel cancer (6% lifetime risk) (Ford *et al*, 1994). Male BRCA2 carriers have an estimated 6% risk of breast cancer by age 70 (Easton *et al*, 1997) and a 10% risk of prostate cancer (Edwards *et al*, 2003). There may also be increased risks of other cancers, although the absolute risks are smaller.

The literature on psychosocial aspects of BRCA1/2 genetic testing focuses mainly on highly researched cohorts involved in original linkage studies (Croyle *et al*, 1997; Lerman *et al*, 1997; Smith *et al*, 1999). There are few large clinical studies and few include men (Lodder *et al*, 2001b; Schwartz *et al*, 2002). Where the psychological impact of BRCA1/2 testing on men is reported, the numbers are small and limit interpretation. Most studies assessing the short-term psychological impact of testing for BRCA1/2 (usually within the month post-genetic test) indicate that noncarriers experience a reduction in anxiety, whereas carriers appear to derive few psychological benefits (Lerman *et al*, 1996; Croyle *et al*, 1997; Lodder *et al*, 2001a; Schwartz *et al*, 2002). One meta-analysis (Braithwaite *et al*, 2004) concluded that there are no adverse psychosocial sequelae to genetic counselling, but the long-term impact of genetic testing has not been reviewed due to insufficient studies. Only one study has described the psychological impact of testing over the following year, and this was in a small clinical cohort and cancer worry was not assessed (Meiser *et al*, 2002). Data suggest that noncarriers derive psychological benefits from genetic testing, such as a reduction in worries about developing cancer. Gene carriers, however, continue to experience cancer-related distress, which is highest in the month following the genetic test.

Women with a family history of breast/ovarian cancer tend to overestimate their risk of developing cancer (Evans *et al*, 1993; Watson *et al*, 1999) and cancer-related distress (Lerman *et al*, 1995) may interfere with the comprehension of individualised genetic risk information. If genetic testing is to provide benefits, it is important to ensure that those offered testing understand risk information and the advice given, so that informed choices can be made in relation to risk management.

A primary motivation for many healthy women to proceed with predictive genetic testing involves reduction of risk, the risk of developing cancer or dying of cancer (Lodder *et al*, 1999). Research evidence is limited regarding management of risk once carrier status is known. Data have been reported on risk management *intentions* in the month following BRCA1/2 testing (Lerman *et al*, 1996; Lodder *et al*, 2001a). Both studies report similar risk management intentions in terms of risk-reducing oophorectomy, with about one-third of gene carriers intending to have their ovaries removed. Intentions to have risk-reducing mastectomy varied considerably, 17% (two out of 12) (Lerman *et al*, 1996) *vs* 40% (10 out of 25) (Lodder *et al*, 2001a). However, 1 month post-testing does not provide a long-enough period in which to assess the risk management implications of genetic testing in the longer term. It takes time for these services to be put in place and actual behaviour may differ from intended behaviour.

Individuals identified as BRCA1/2 gene mutation carriers can potentially reduce the risk associated with breast/ovarian cancer by electing to undergo risk-reducing surgery, chemoprevention, or regular surveillance to detect cancer at an early stage when it is more treatable (Hendrick *et al*, 1997; Hartmann *et al*, 2001; Rebbeck *et al*, 2002). Studies suggest that surgery (bilateral mastectomy or oophorectomy) reduces the risk of breast cancer by 90% (Hartmann *et al*, 2001) and ovarian cancer by up to 95% (Rebbeck *et al*, 2002). The effectiveness of chemoprevention (e.g. Tamoxifen and other selective oestrogen receptor modulators (SERMS)) for gene carriers is unclear (Eeles and Powles, 2000). The tendency for a significant proportion of gene carriers (particularly BRCA1) to have oestrogen-receptor-negative breast cancer suggests limited efficacy of SERMS in the treatment of breast cancer in this group (Lakhani *et al*, 2002). There is limited evidence on the efficacy of mammography in pre-menopausal women, although some benefit is likely for those over age 40 (Lalloo *et al*, 1998; Moller *et al*, 2002). Lack of efficacy may be due to screening trials assessing younger age groups (i.e. 40–49 years) being underpowered rather than limits to the breast X-ray technique. Surveillance using magnetic resonance imaging (MRI) may be more sensitive than mammography in this younger age group, but there is inadequate evidence at present to justify routine use (Stoutjesdijk *et al*, 2001). There is also limited evidence on the rates of breast biopsy following MRI and, due to the limited specificity of MRI, benign breast disease and cyclical changes may be treated as suspicious in premenopausal women, leading to increased invasive diagnostic procedures (Warren *et al*, 2002). Trials are underway in the UK to establish the efficacy of ovarian screening in women at increased risk of ovarian cancer due to family history over age 35 years (UK Familial Ovarian Cancer Screening Study) and in the general population over 50 years of age (UK Trial of Ovarian Cancer Screening).

It is important to evaluate the use of genetic information by the insurance industry. Patients have declined genetic testing due to concerns about insurance discrimination (Morrison *et al*, 1999). Patients have also experienced problems with insurance discrimination (Morrison *et al*, 2000). There is currently a moratorium on using genetic test results in calculating insurance premiums (Morrison, 2001). Specifically, the moratorium applies to life insurance policies up to £500 000 and critical illness, long-term care insurance and income protection up to £300 000 for each type of policy. The use of negative results is encouraged by insurers where a geneticist can confirm the relevance of the result. Therefore, we asked study participants to report whether or not they had experienced any insurance-related problems following their predictive genetic test.

Due to insufficient evidence to support the introduction of BRCA1/2 genetic testing into the clinical context, this prospective study with follow-up to 1 year post-test was undertaken throughout the UK between 1996 and 2003 (follow-up continuing to 3 years). The present study includes men and women unaffected by cancer (i.e. no diagnosed cancer) attending nine centres that currently undertake the majority of BRCA1/2 testing in clinical cohorts in the UK. The study aims to document any psychological morbidity related to genetic testing, ascertain cancer risk perceptions and examine the ongoing risk management in BRCA1/2 carriers and noncarriers in the year following genetic testing. Clarifying whether insurance discrimination has been experienced as a result of genetic testing is a further aim. Comparisons will be made between carriers and noncarriers, men and women. Three key questions are addressed:

What mental health problems exist in men and women and how are they affected both short and long term by BRCA1/2 genetic testing?

Do female participants understand their risks of developing cancer following genetic testing?

How does BRCA1/2 genetic testing impact upon risk management for women?

## MATERIALS AND METHODS

Participants were unaffected by breast or ovarian cancer at the time of study entry, from families with an identified BRCA1/2 mutation, and a 50% (25% if an intervening relative had died) risk of inheriting a BRCA1/2 mutation. Three individuals declined participation, six declined genetic testing following genetic counselling and did not receive baseline questionnaires, nine did not return the study questionnaire, leaving a total of 298 out of 315 (97%) with completed baseline questionnaires (227 females, 71 males).

### Procedure

Using a prospective design, adults from nine clinical genetic centres participated. Trent multi-centre research ethics committee and all local research ethics committees approved the study. The consultant geneticist or genetic associate/nurse recruited participants via clinics between 1996 and 2000. Study entry followed the genetic counselling consultation prior to the consultation at which blood was drawn for genetic analysis. Written consent was obtained.

The baseline questionnaire, given at the clinic prior to genetic testing, was returned directly to the data management centre at the Institute of Cancer Research, UK. These baseline data are reported in detail elsewhere (Foster *et al*, 2002). Follow-up assessment began once clinicians informed the data centre that participants had received their genetic test result and questionnaires were mailed at 1, 4 and 12 months.

### Measures

Baseline demographic data included age, level of education, marital status, number and age of biological children, and social class. Mutation carrier status was collected from clinics at the end of the study. Measures selected for validity, reliability and prior application within genetics research included:

#### General health questionnaire 28 (GHQ28)

(Goldberg and Hillier, 1979): this 28-item measure screens for psychiatric disorder (cases) in nonpsychiatric populations (clinical case cutoff score of ⩾10 (binary scoring) was used (Hopwood *et al*, 1998)). Male and female participants were assessed at baseline and follow-up.

#### Cancer worry scale-revised – CWS-R

(Watson *et al*, 1999): this six-item scale assesses the degree of worry about developing cancer using a four-point rating from ‘Not at all or rarely’ to ‘Almost all the time’. A total score on the CWS-R ranges from 6 to 24. A high score indicates greater worry. No clinical cutoffs are currently available. The follow-up data yield an alpha coefficient of 0.87. Female participants were assessed at baseline and follow-up. At the time the study was designed, knowledge regarding additional risks to men due to BRCA1/2 mutations was limited; therefore, it was not deemed appropriate to ask men about their risk of developing cancer.

#### Impact of event scale IES

(Horowitz *et al*, 1979): measures the level of distress in response to a specific traumatic event. A modified 15-item version (female participants only) assesses specific thoughts about risk of cancer over the last 7 seven days (Kash *et al*, 1992; Watson *et al*, 1999), with a high score indicating more frequent intrusive/avoidant thoughts about risk of cancer.

#### Risk perception

At baseline, the perceived risk of developing breast/ovarian cancer (female participants only) was assessed on a three-point scale (‘Not very likely’ to ‘Very likely’) or as a stated percentage or odds ratio. Knowledge of general population risk for breast/ovarian cancer was assessed. At baseline and 1-month follow-up, the perceived risk relative to the general population was rated on a five-point scale (‘Very much lower than average’ to ‘Very much higher’). At 1 and 12 months, women were asked ‘What risk figure have you been given for developing breast/ovarian cancer?’, with six response options (‘Less than average woman’, ‘Same as average woman’, ‘50 : 50 chance’, ‘85% chance’, ‘Other – please specify’, ‘Can’t remember’). Men were asked if they felt that they were at increased risk of developing cancer (12-month follow-up only).

#### Risk management

Women reported on the uptake of risk management options since their genetic test, including mammography, Tamoxifen use, bilateral risk-reducing mastectomy (BRMx), bilateral risk-reducing oophorectomy (BROx), ovarian ultrasound (Ov US), clinical examination of the breasts by a doctor (CBE), breast self-examination (BrSE) or any other screening for cancer. Frequencies of BrSE and breast/ovarian biopsy rates were recorded.

At all follow-ups, data were collected on number of GP visits since genetic testing to discuss the result or test-related concerns, and rates of medication for depression, worry or sleeplessness.

#### Insurance issues

Information was requested on any difficulties with life or health insurance following their genetic test at 12 months. Participants were not asked if they had declared the test result to their insurer.

### Statistical method

The association between categorical variables is examined using Fisher's exact test or the *χ*^2^ test, with Yates correction where appropriate. For ordered categorical variables, the Mann–Whitney test for trend was used. Age was analysed as a continuous variable and, in addition, participants were divided into three age groups (<35, 35–49 and ⩾50 years) chosen to reflect the variations that might occur in risk management in these age groups especially in relation to screening by mammography. Women under 35 years are unlikely to receive a mammogram and women over 50 years receive regular mammograms as part of the UK National Screening Programme. Where the <35 and 35–49 year age groups behave in a similar fashion compared to the >50s, the two younger groups are reported as one (<50). Scores from the GHQ28, IES and CWS-R are treated as continuous variables. Normality is tested using the Kolmogorov–Smirnov statistic and all scores are shown to be non-normally distributed. Scores are summarised using median and range. Differences between groups (carriers and noncarriers; men and women; age groups) were assessed using the Kruskal–Wallis (KW) test. Changes from baseline were assessed by the signed rank test. Owing to the relative invariance of the median, the mean scores are used in the figures. Participants with missing data are omitted from the respective analyses.

## RESULTS

A total of 298 individuals completed baseline questionnaires (including genetic test decliners) and 285 received a genetic test result and follow-up questionnaires; 100 (35%) carriers and 185 (65%) noncarriers. In all, 261 participants (92%) returned follow-up questionnaires overall; 91 (35%) carriers and 170 (65%) noncarriers. A total of 228 (80%) completed questionnaires were returned at 1 month (15 were not sent due to administrative error, 1 further questionnaire was not sent due to withdrawn participation in the study), 242 (85%) at 4 months (four were not sent due to administrative error) and 235 (82%) at 12 months. The proportion of carriers and male participants at baseline was the same for responders at follow-up. There were no differences between responders and nonresponders in terms of baseline demographics or psychosocial characteristics. Data for individuals who declined genetic testing at the time of study entry are reported elsewhere (Foster *et al*, 2004).

In all, 23% (*N*=59) at follow-up were male, 84% married or cohabiting, 41% college or university educated, and most men (73%) and women (67%) were currently employed. The median age for women was 41 (23–72 years) and 50 (22–86 years) for men. In all, 45 women and 10 men were <35 years; 107 women and 18 men were 35–49 years; 48 women and 31 men were ⩾50 years. Among gene carriers, 19 females and six males were <35 years, 42 females and nine males were 35–49, and five females and 10 males were ≥50. Most had children (87%) and the median age of offspring was 19 years (0–50 years). Altogether, 85% described themselves as Caucasian; 67% of the women and 58% of the men were noncarriers. Of the 91 carriers, 64 (70%) had BRCA1, 26 (29%) BRCA2 and one (1%) both BRCA1 and BRCA2 genetic faults.

Participants at each centre (responding at follow-up) were compared on demographic variables. Three centres (St Mary's Hospital Manchester, Royal Marsden Hospital London/Sutton and Princess Ann Hospital, Southampton) accounted for 82% of the participants; two smaller centres recruited only women (Cambridge and Birmingham). There were no differences in comparisons across the three larger centres, except that the London/Sutton patients had a marginally higher level of educational achievement than those from Southampton (*P*=0.06). Participants (*n*=47) from the six smaller centres are younger (*P*<0.005).

### Mental health

There were no baseline differences between carriers and noncarriers on mental health measures (GHQ *P*=0.7, CWS *P*=0.9).

### GHQ28

There were no significant changes from baseline in GHQ scores in male carriers (1 month, *P*=0.4; 4 months, *P*=0.6; 1 year, *P*=0.2) or noncarriers (1 month, *P*=0.1; 4 months, *P*=0.1; 1 year, *P*=0.7).

**Figure 1:**
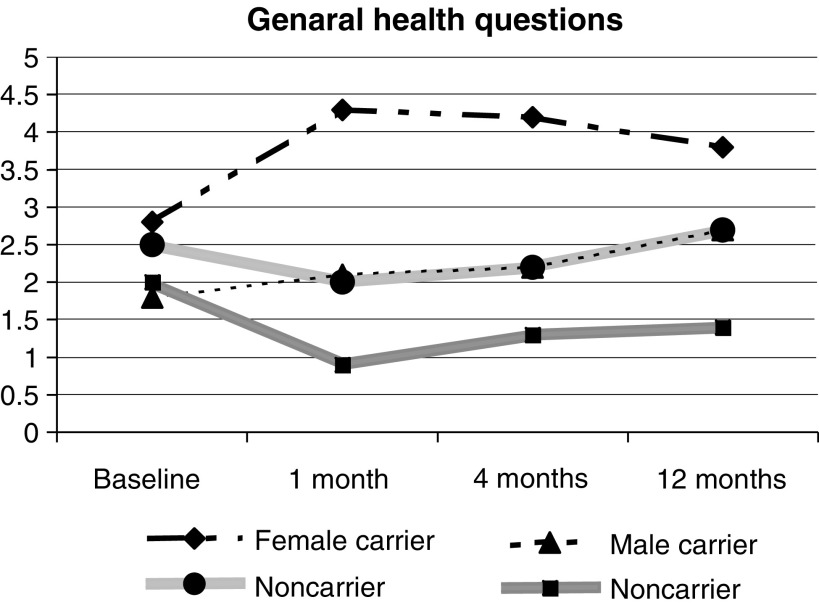
Changes in GHQ28 from baseline in the 12 months following genetic testing.

Female carriers had significantly higher GHQ total symptom scores than noncarriers at 1 month (1 (0 : 26) *vs* 0 (0 : 20) *P*=0.009) and 4 (1 (0 : 20) *vs* 0 (0 : 22) *P*=0.005) months, but not at 12 months (0 (0 : 20) *vs* 0 (0 : 25) *P*=0.4), and had higher GHQ scores at 1 (0 (−8 : +18), *P*=0.02) and 4 months (0 (−18 : +19), *P*= 0.02) compared to baseline, but this had returned to baseline levels at 1 year (0 (−15 : +23), *P*=0.6). This increase was only significant in the 35–49 age group at 1 month (0 (−4 : +18); *P*=0.02). Low numbers may be the reason for not seeing a difference in the over 50 group, where there were only five carriers (Figure 1

).

### Cancer worry scale-revised (females only)

**Figure 2:**
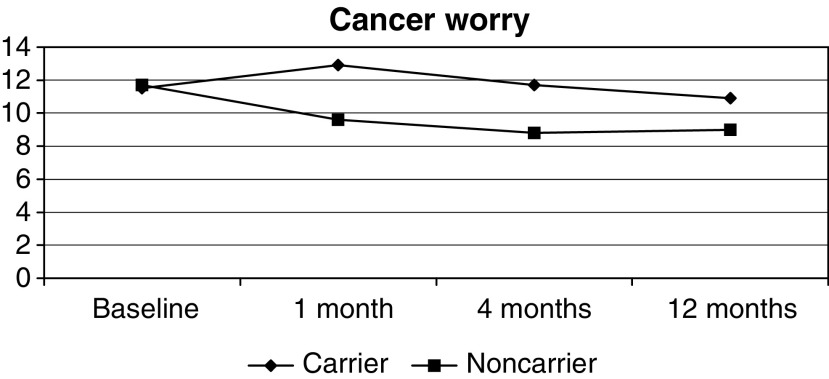
Mean cancer worry scores (CWS-R) in the year following disclosure of BRCA1/2 carrier status (women only).

Carriers reported (Figure 2

) significantly higher levels of cancer worry than noncarriers at all follow-up points (1 month: 13 (6 : 21) *vs* 9 (( : 20) *P*<0.001; 4 months: 11 (6 : 21) *vs* 9 (6 : 16), *P*<0.001; 1 year: 10 (6 : 22) *vs* 9 (6 : 16), *P*=0.003). There was an increase in worry for carriers at 1 month (+1 (−10 : +13), *P*=0.001) compared to baseline and a decrease at all time points in noncarriers (1 month: −2 (−13 : +6), *P*<0.001; 4 months: −2 (−12 : +3), *P*<0.001; 1 year: −2 (−14 : +5), *P*<0.001). At 1 month after genetic test result disclosure, cancer worry in carriers was higher in the younger age groups (<35: 13 (6 : 19); 35–49: 13 (7 : 21)) than in the ⩾50 group (9.5 (7 : 11)) *P*=0.03), but this age effect was not significant at 1 year. Among noncarriers, the reduction in cancer worry was greater at each time point in the younger age group (<50) (1 month: −2 (−13 : +6) *vs* 0 (−8 : +3), *P*=0.001; 4 months: −3 (−12 : +3) *vs* −2 (−8 : +3), *P*=0.001; 1 year: −3 (−14 : +4) *vs* −1 (−8 : +5), *P*<0.001). In all, 39, 24 and 18% of female carriers worried about developing cancer ‘frequently’ or ‘constantly’ at 1, 4 and 12 months, respectively, compared to just 5, 7 and 5% of noncarriers (*P*<0.01 at all time points). Also, 22, 20 and 10% said that their worry was a ‘definite’ or ‘severe’ problem at all time points compared with 3, 2 and 2% of noncarriers (*P*<0.01).

### Impact of event scale (females only)

There was a significant difference between carriers and noncarriers at all follow-up points for avoidant (1 month, *P*<0.001; 4 months, *P*<0.001; 1 year, *P*<0.03) and intrusive thoughts (1 month, *P*< 0.001; 4 months, *P*<0.001; 1 year, *P*=0.03). Gene carriers showed no significant increase from baseline in avoidant thoughts, but there was an increase in intrusive thoughts at 1 month only compared to baseline (*P*<0.001).

In total, 20 female carriers, six female noncarriers and three male carriers sought professional help/advice in the year following receipt of their genetic test result. Women were asked if they had taken any medication for depression, worry or sleeplessness. Of the carriers (noncarriers shown in brackets), nine (3) had taken medication for depression, seven (10) for worry, four (3) for sleeplessness and one (1) for unspecified reasons.

### Risk perception

**Figure 3:**
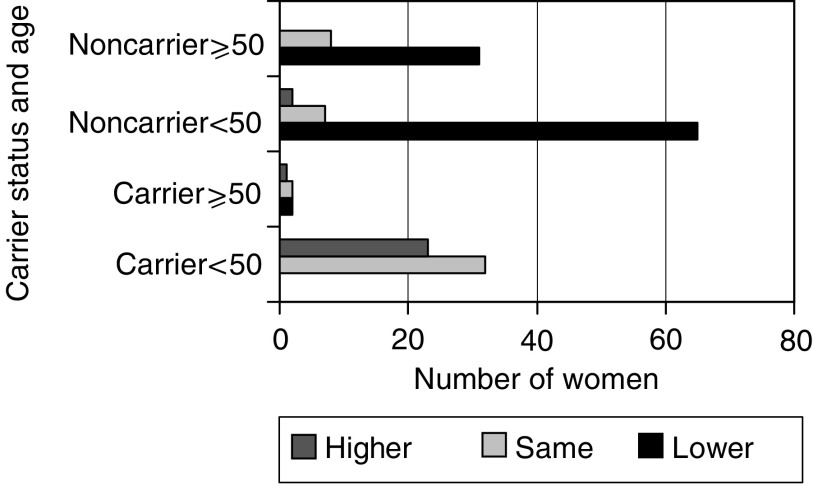
Change in risk perception 1 month following the genetic test result.

At baseline, most women thought that they were at a higher than average risk of breast (88%) and ovarian cancer (69%). (One woman who thought her risk of breast cancer was much lower than average had already had a BRMx.) There was no difference between carriers and non-carriers at baseline (breast cancer *P*=0.4; ovarian cancer *P*=0.5; MW_Trend_). By 1 month follow-up, risk perception is lower in noncarriers and higher in gene mutation carriers compared to baseline (see Figure 3

). There is evidence that younger females have increased their perception of risk (<50 years; *P*=0.04; MW) in line with their carrier status. Scheduled BROx post-testing in the gene carriers has the effect of lessening the increased risk perception (*P*<0.05; MW) when measured 1 month following disclosure of the test result.

At 1 year, 88% of noncarriers rated their risk of breast cancer as the ‘same as the average woman’. Eight women (7%) said that they had a less than average risk, two thought they had a slightly higher than average risk, one woman thought she was at high risk. Among carriers, most (71%) thought that they had an 85% chance of developing breast cancer. All other carriers thought they were at increased risk. No carriers thought that they had an average or less than average risk. When asked at baseline if they felt at increased risk of developing other cancers, 25 female carriers said they had an increased risk of gastro-intestinal cancer, nine lung cancer, five cervical/endometrial and 10 other cancers of unspecified type. Nine male carriers said they were at increased risk of gastro-intestinal cancer, two lung cancer, 13 prostate cancer and three unspecified cancers.

### Risk management

Two women had BRMx (both noncarriers) and 24 BROx (five gene carriers and 19 noncarriers) prior to genetic testing.

Table 1

**Table 1:** Risk management procedures over the 12 months from genetic testing according to age (⩾50 or <50 years) and carrier status (percentage of women)

	**Carrier (<50)**	**Carrier (⩾50)**	**Noncarrier (<50)**	**Noncarrier (⩾50)**
Mammogram	75	100	24	41
CBE	65	100	26	12
Tamoxifen	2	20	1	6
BRMx	28	0	0	0
Ov US	61	40	5	12
BROx	32	40	2	2
Breast biopsy	3	20	4	5
Ovarian biopsy	7	0	0	2
BrSE	96	100	95	88

illustrates the percentage of women undergoing risk management procedures in the 12 months following genetic testing. In all, 20 carriers (31%) had BROx following their genetic test result, three noncarriers had their ovaries removed following testing as part of a therapeutic hysterectomy. In all, 42% (20 out of 48) of gene carriers with children (mean 2.5 children; range 1–4) had BROx following testing compared to those (zero out of 14) without children (*P*<0.01). There was marginal evidence that those having BROx were older; mean age 41 years (range 29–59) *vs* 37 (range 25–64) (*P*=0.06). There were no BRMxs during the year following predictive testing in noncarriers, but 17 (28%) gene carriers had this surgery post-genetic testing. All of these women were <50 years and four were aged <35. The rate of BRMx among gene carriers varied from centre to centre; 42% (10 out of 24) of Manchester patients, 36% (four out of 11) of Royal Marsden Hospital patients, 7% (one out of 14) of Southampton patients and 13% (two out of 15) elsewhere. Six women had both BROx and BRMx. There was no evidence that having risk-reducing surgery (*N*=30) reduced the levels of cancer worry, avoidant or intrusive (all *P*>1) thoughts in gene carriers by 12 months follow-up. The numbers are small and this effect should be interpreted with caution.

Three women identified as noncarriers subsequently remained in the Tamoxifen chemoprevention trial (IBIS 1 2003) compared to two carriers.

Rates of mammography were significantly different (*P*<0.001) between carriers (92%) and noncarriers (30%) at 12 months, where there had previously been no difference at baseline (*P*>1). Noncarriers who were older (⩾50) were marginally more likely to have had a mammogram since their test result than younger women (41 *vs* 24%; *P*=0.07; Fisher), since they would be included in the national screening programme and continue to have increased risk due to age. There were no other age differences.

A similar effect was noted for rates of CBE (90% in carriers *vs* 89% in noncarriers; *P*=0.04) and ovarian US (59% in carriers *vs* 8% in noncarriers; *P*<0.001). There were no differences in risk management options undertaken by BRCA1 and BRCA2 carriers.

These changes in risk management are all in the expected direction, with a reduction for noncarriers at 12 months follow-up. There was no evidence that risk management uptake reduced cancer worry at 1 year (*P*>0.1 for all tests; carriers and noncarriers). Not surprisingly, carriers who had a breast biopsy during the year (three patients) had an increase in cancer worry (*P*=0.03).

In all, 87% of women reported practising BrSE at baseline. There was no difference in baseline frequency between carriers and noncarriers (*P*=0.5). At 1 year, 91% of carriers and 84% of noncarriers reported practising BrSE (*P*=0.02). Since almost all women reported practising BrSE, it is not possible to demonstrate a relationship with cancer worry. In total, 29% of women reported increased frequency of BrSE following testing, with no difference between carriers and noncarriers (35 *vs* 28%; *P*>1); 26% of women reported that they had decreased the frequency of BrSE and this was more common among noncarriers than carriers (12 *vs* 31%; *P*<0.01). Breast self-examination is recommended on a monthly basis. Most carriers reported performing BrSE monthly (33%) or more frequently (39%). However, 18% of carriers performed BrSE less frequently and 10% never. In comparison, 36% of noncarriers reported performing BrSE monthly, 21% more frequently, 27% less frequently and 16% never. At 1 year, 80% of gene carriers and 62% of noncarriers had read leaflets on breast awareness (*P*=0.02). Most women (82% of carriers and 81% of noncarriers; *P*=1.0) had been shown how to examine their breasts.

Altogether, 52% of female carriers (43% noncarriers) and 44% of male carriers (44% noncarriers) reported having done something else to help them stay healthy and/or avoid cancer. They reported changes in diet (33% female carriers *vs* 36% NC; 24% male carriers *vs* 29% NC) and exercise (17% female carriers *vs* 19% NC; 20% male carriers *vs* 9% NC). Reducing/stopping smoking was reported by 8% female carriers *vs* 1% NC (*P*=0.04) and 4% male carriers *vs* 13% NC. Female carriers were more likely than female noncarriers to report taking vitamins (*P*=0.02). There were no differences between male carriers and noncarriers.

### Insurance discrimination (females only assessed)

Five gene carriers reported difficulties in obtaining life insurance following testing (one was made ‘A-risk’, three were refused insurance and one could only find one company willing to insure her), eight (two women had both life and health insurance problems) gene carriers had difficulties obtaining health insurance (one was made ‘A risk’, one company will not pay for anything until the woman gets cancer, one was refused critical illness cover on her endowment policy, one was refused income protection, in three cases the company refused to pay for risk-reducing surgery) and two women had their premiums increased (not known which insurance)). Thus, in total, 13 female carriers (20%) experienced some form of insurance problem during the year following genetic test. In addition, one woman who was a noncarrier had difficulties with life insurance, where the company involved refused to remove a loaded policy premium. We do not have further details regarding why these individuals experienced problems with their insurance.

## DISCUSSION

This study is unique in clarifying what happens during the year following BRCA1/2 genetic testing now it is becoming routine clinical practice. Our sample includes a substantial group of men, most of whom are over 50 years and have children. Male participants did not report poorer general mental health scores following genetic testing. For female gene carriers, worry and mental health problems were highest during the month following receipt of the genetic test result. These adverse reactions gradually returned to pre-test baseline levels over the following 12 months. For noncarriers, there was an immediate and sustained lowering of cancer-related worry from baseline levels. In this respect, noncarriers derived most benefit to their mental health, whereas carriers, after an initial increase, continued to experience the same level of worry as pre-testing. This reflects the findings from other studies (Lerman *et al*, 1997; Reichelt *et al*, 1999). Among female carriers, younger (<50 years) women were more likely to report higher levels of cancer worry in the month following testing, but they reported similar levels of worry later in the year. A significant proportion of these women rated their worry as severe or problematic. It is clear that appropriate services should be put in place to identify individuals most likely to need extra psychological support following the test result.

To optimise health benefits, individuals should understand risk information presented in genetic counselling, so that informed decisions can be made regarding risk management. It is reassuring that most noncarriers reported being at a lower risk than at baseline 1 month following testing. This was maintained a year after disclosure of carrier status. Younger female carriers were more likely to report increases in perceived risk of developing cancer in the year following testing compared to their baseline responses, but as a group their perceived risk was generally unchanged from baseline. Women who had risk-reducing surgery (mastectomy and/or oophorectomy) reported a reduction in their risk of developing cancer.

Given that one aim of predictive testing for BRCA1/2 is to reduce mortality by regular surveillance or surgery, it is important to clarify concerns and support those having difficulty engaging in appropriate risk management options. In terms of risk management, this study provides some useful data on the cohort of carriers and noncarriers, which has not been reported elsewhere. Carriers were likely to engage in risk management, noncarriers were less likely to engage in risk management, as expected. However, although noncarriers do not have a BRCA1/2 mutation that predisposes them to breast/ovarian cancer at a younger than expected age, they are still at population risk. Sporadic breast/ovarian cancers are most likely to occur after the menopause; therefore, it is possibly of concern that a significant proportion of noncarriers report reducing the frequency of BrSE following their test result, and this is generally in older women. It is not clear, however, if this merely represents a drop in BrSE to that of the general female population. Nonetheless, this may be a potential area for additional information to noncarriers as they remain at population risk for breast cancer and this risk increases with age. In all, 41% of female noncarriers over 50 years had received a mammogram in the year following testing. This would appear in keeping with the 3-yearly interval of the UK national breast-screening programme. Nevertheless, it is important to ensure that women do not interpret a negative genetic testing as eliminating their cancer risk. It may be that 12 months is too short a time period to fully assess the overall impact of testing on participation in screening programmes or uptake of risk-reducing surgery. A further assessment at 3 years following testing is currently ongoing and these data will be reported in due course.

It is striking that a number of women had risk-reducing surgery (oophorectomy or bilateral mastectomy) prior to genetic testing. The majority of these were subsequently identified as noncarriers. Most will have had this surgery prior to the availability of genetic testing. For the two noncarriers who had BRMx and the 19 who had BROx, this surgery might have been avoided if genetic testing had been available to them when surgery was being contemplated. In this respect, genetic testing will likely save some women unnecessary surgery and reduce costs to the NHS and the women themselves. On this basis, risk-reducing surgery should preferably not be offered to unaffected women until the possibility of having a genetic test to clarify her status has been fully explored.

There was no evidence to suggest that having risk-reducing surgery reduced the levels of cancer worry, or avoidant/intrusive thoughts 1 year following testing. This merits further investigation as worry levels in gene carriers remained high over the year following genetic testing. It may be the case that some women had only just completed their surgery on either their breasts or ovaries (BRMx or BROx), and had not yet had intended surgery on the other organ still at risk. It would be helpful to ascertain why there was a residual high level of cancer-related worry where women had risk-reducing surgery, as these data would appear to contradict other available evidence (Hatcher *et al*, 2001).

Three noncarriers chose to remain in the IBIS (Tamoxifen) chemoprevention trial in the year following genetic testing, and could have been discharged from this study on the basis that they no longer met criteria for inclusion.

Few individuals regretted their decision to have a predictive genetic test and most were pleased. However, 20% of female gene carriers experienced some form of insurance discrimination during the year following genetic testing, which is high, considering that some will not have applied for insurance in the year following testing. We do not have more detailed information on insurance-related problems at this stage, but we are collecting additional information at 3-year follow-up. Even one case of discrimination gives rise to worries about the use of genetic information by the insurance industry. Given that there is currently a moratorium on using genetic testing results in calculating insurance premiums (Morrison, 2001), it is of concern that so many women reported experiencing problems.

Study participants were recruited from nine centres offering genetic testing for BRCA1/2 between 1996 and 2000. Three centres recruited most participants as they had an established clinical service in place at the time the study began. Far fewer individuals were recruited from other centres; therefore there may be some differences in participant responses that are a factor of the clinical service. However, these data provide an important overview of patient experiences in the period following testing in a group of centres, providing a significant proportion of genetic testing during the recruitment period. Nonresponders at follow-up did not have significantly different worry, mental health or risk perception scores at baseline, and did not differ significantly in terms of demographic characteristics. We do not know the carrier status of nonresponders.

In summary, these data illustrate that while general mental health and cancer-related worry are reduced in noncarriers in the year following testing, carriers receive few psychological benefits from discovering their carrier status – particularly among younger women. In the future, genetic testing is likely to become more accessible and more rapid. Pre-test counselling should continue to be provided. Younger women appear to be the most vulnerable group. Many carriers in this cohort, especially younger women, experienced increased levels of distress in the short term following disclosure of their test result, which then returned to pre-test levels. In addition to the absence of psychological benefits of testing in younger female carriers, there is no clear evidence regarding the medical or psychological benefits of continued breast/ovarian surveillance in this group; however, this requires further investigation. It is important that genetic counselling and support is also available in clinics following disclosure of the test result in order to address concerns, receive information to make informed choices and continue to be supported both medically and psychologically. The development of carrier clinics may improve access to information and support. Regular updates of new options via leaflets, information packs, the Internet or family study days will keep patients up to date. Mental health liaison services should also be made available for those experiencing persistent distress.

## References

[bib1] Braithwaite D, Emery J, Walter F, Provost AT, Sutton S (2004) Psychological impact of genetic counseling for familial cancer: a systematic review and meta-analysis. J Natl Cancer Inst 96: 122–133 14734702 10.1093/jnci/djh017

[bib2] Croyle RT, Achilles JS, Lerman C (1997) Psychologic aspects of cancer genetic testing: a research update for clinicians. Cancer 80: 569–575 11657060 10.1002/(sici)1097-0142(19970801)80:3+<569::aid-cncr6>3.3.co;2-9

[bib3] Easton D, Steele L, Fields P, Ormiston W, Averill D, Daly P, McManus R, Neuhausen S, Ford D, Wooster R, Cannon-Albright L, Stratton M, Goldgar D (1997) Cancer risks in two large breast cancer families linked to BRCA2 on chromosome 13q12–13. Am J Hum Genet 61: 120–128 9245992 10.1086/513891 PMC1715847

[bib4] Edwards SM, Kote-Jarai Z, Meitz J, Hamoudi R, Hope Q, Osin P, Jackson R, Southgate C, Singh R, Falconer A, Dearnaley D, Ardern-Jones A, Murkin A, Dowe A, Kelly J, Williams S, Oram R, Stevens M, Teare DM, Ponder BAJ, Gayther SA, Cancer Research UK and British Prostate Group UK Familial Prostate Cancer Study Collaborators and British Association of Urological Surgeons Section of Oncology, Easton DF, Eeles RA (2003) Two percent of men with early-onset prostate cancer harbor germline mutations in the BRCA2 gene. Am J Hum Genet 72: 1–12 12474142 10.1086/345310 PMC420008

[bib5] Eeles R, Powles TJ (2000) Chemoprevention options for BRCA1 and BRCA2 mutation carriers. J Clin Oncol 18: 93S–99S 11060334

[bib36] Evans DGR, Burnell LD, Hopwood P, Howell A (1993) Perception of risk in women with a family history of breast cancer. Br J Cancer 67: 612–614 8439512 10.1038/bjc.1993.112 PMC1968271

[bib6] Ford D, Easton D, Bishop DT, Narod S, Goldgar D, the Breast Cancer Linkage Consortium (1994) Risks of cancer in BRCA1 mutation carriers. Lancet 343: 692–695 7907678 10.1016/s0140-6736(94)91578-4

[bib7] Ford D, Easton D, Stratton M, Narod S, Goldgar D, Devilee P, Bishop D, Weber B, Lenoir G, Chang-Claude J, Sobol H, Teare M, Struewing J, Arason A, Scherneck S, Peto J, Rebbeck T, Tonin P, Neuhausen S, Barkardottir R, Eyfjord J, Lynch H, Ponder B, Gayther S, Birch J, Lindblom A, Stoppa-Lyonnet D, Bignon Y, Borg A, Hamann U, Haites N, Scott R, Maugard C, Vasen H, Seitz S, Cannon-Albright L, Schofield A, Zelada-Hedman M, the Breast Cancer Linkage Consortium (1998) Genetic heterogeneity and penetrance analysis of the BRCA1 and BRCA2 genes in breast cancer families. Am J Hum Genet 62: 676–689 9497246 10.1086/301749 PMC1376944

[bib8] Foster C, Evans D, Eeles R, Eccles D, Ashley S, Brooks L, Cole T, Cook J, Davidson R, Gregory H, Mackay J, Morrison P, Watson M (2004) Non-uptake of predictive genetic testing for BRCA1/2 among relatives of known carriers: attributes, cancer worry and barriers to testing. Genet Test 8: 23–29 15140371 10.1089/109065704323016003

[bib9] Foster C, Evans DGR, Eeles R, Eccles D, Ashley S, Brooks L, Davidson R, Mackay J, Morrison PJ, Watson M (2002) Predictive testing for BRCA1/2: attributes, risk perception and management in a multi-centre clinical cohort. Br J Cancer 86: 1209–1216 11953874 10.1038/sj.bjc.6600253 PMC2375339

[bib10] Goldberg DP, Hillier VF (1979) A scaled version of the General Health Questionnaire. Psychol Med 9: 139–145 424481 10.1017/s0033291700021644

[bib11] Hartmann LC, Sellers TA, Schaid DJ, Frank TS, Soderberg CL, Sitta DL, Frost MH, Grant CS, Donohue JH, Woods JE, McDonnell SK, Vockley CW, Deffenbaugh A, Couch FJ, Jenkins RB (2001) Efficacy of bilateral prophylactic mastectomy in BRCA1 and BRCA2 gene mutation carriers. J Natl Cancer Inst 93: 1633–1637 11698567 10.1093/jnci/93.21.1633

[bib12] Hatcher M, Fallowfield L, A'Hern R (2001) The psychosocial impact of bilateral prophylactic mastectomy: prospective study using questionnaires and semistructured interviews. Br Med J 322: 76 11154619 10.1136/bmj.322.7278.76 PMC26594

[bib13] Hendrick R, Smith R, Rutledge, Smart C (1997) Benefit of screening mammography in women aged 40–49: a new meta-analysis of randomized controlled trials. J Natl Cancer Inst Monogr 22: 87–92 10.1093/jncimono/1997.22.87 9709282

[bib14] Hopwood P, Keeling F, Long A, Pool C, Evans G, Howell A (1998) Psychological support needs for women at high genetic risk of breast cancer: some preliminary indicators. Psycho-Oncology 7: 402–412 9809331 10.1002/(SICI)1099-1611(1998090)7:5<402::AID-PON317>3.0.CO;2-X

[bib15] Horowitz M, Wilner N, Alvarez WW (1979) Impact of events scale: a measure of subjective stress. Psychosomatic Med 41: 209–218 10.1097/00006842-197905000-00004 472086

[bib16] Kash KM, Holland JC, Halper MS, Miller DG (1992) Psychological distress and surveillance behaviours of women with a family history of breast cancer. J Natl Cancer Inst 84: 24–30 1738170 10.1093/jnci/84.1.24

[bib17] Lakhani S, Van De Vijver M, Jacquemier J, Anderson T, Osin P, McGuffog L, Easton D (2002) The pathology of familial breast cancer: predictive value of immunohistochemical markers estrogen receptor, progesterone receptor, HER-2, and p53 in patients with mutations in BRCA1 and BRCA2. J Clin Oncol 20: 2310–2318 11981002 10.1200/JCO.2002.09.023

[bib18] Lalloo F, Boggis CRM, Evans DGR, Shenton A, Threlfall AG, Howell A (1998) Screening by mammography women with a family history of breast cancer. Eur J Cancer 34: 937–940 9797712 10.1016/s0959-8049(98)00005-7

[bib37] Lerman C, Lustbader E, Rimer B, Daly M, Miller S, Sands C, Balshem A (1995) Effects of individualized breast cancer risk counseling: a randomized trial. J Nat Cancer Inst 87(4): 286–292 7707420 10.1093/jnci/87.4.286

[bib19] Lerman C, Narod S, Schulman K, Hughes C, GomezCaminero A, Bonney G, Gold K, Trock B, Main D, Lynch J, Fulmore C, Snyder C, Lemon SJ, Conway T, Tonin P, Lenoir G, Lynch H (1996) BRCA1 testing in families with hereditary breast-ovarian cancer – a prospective study of patient decision making and outcomes. JAMA (J Am Med Assoc) 275: 1885–1892 8648868

[bib20] Lerman C, Schwartz MD, Lin TH, Hughes C, Narod S, Lynch HT (1997) The influence of psychological distress on use of genetic testing for cancer risk. J Consult Clin Psychol 65: 414–420 9170764 10.1037//0022-006x.65.3.414

[bib21] Lodder L, Frets PG, Trijsburg RW, Meijers-Heijboer EJ, Klijn JGM, Duivenvoorden HJ, Tibben A, Wagner A, van der Meer CA, van den Ouweland AMW, Niermeijer MF (2001a) Psychological impact of receiving a BRCA1/BRCA2 test result. Am J Med Genet 98: 15–24 11426450

[bib22] Lodder L, Frets PG, Trijsburg RW, Tibben A, Meijers-Heijboer EJ, Duivenvoorden HJ, Wagner A, van der Meer CA, Devilee P, Cornelisse CJ, Niermeijer MF (2001b) Men at risk of being a mutation carrier for hereditary breast/ovarian cancer: an exploration of attitudes and psychological functioning during genetic testing. Eur J Hum Genet 9: 492–500 11464240 10.1038/sj.ejhg.5200668

[bib23] Lodder LN, Frets PG, Trijsburg RW, Meijers-Heijboer EJ, Klijn JGM, Duivenvoorden HJ, Tibben A, Wagner A, van der Meer CA, Devilee P, Cornelisse CJ, Niermeijer MF (1999) Presymptomatic testing for BRCA1 and BRCA2: how distressing are the pre-test weeks? J Med Genet 36: 906–913 10593998 PMC1734277

[bib24] Meiser B, Butow P, Friedlander M, Barratt A, Schneiden V, Watson M, Brown J, Tucker K (2002) Psychological impact of genetic testing in women from high-risk breast cancer families. Eur J Cancer 38: 2025–2031 12376208 10.1016/s0959-8049(02)00264-2

[bib25] Miki Y, Swensen J, Shattuck Eidens D, Futreal PA, Harshman K, Tavtigian S, Liu Q, Cochran C, Bennett L, Ding W, Bell R, Rosenthal J, Hussey C, Tran T, McClure M, Frye C, Hattier T, Phelps R, Haugenstrano A, Katcher H, Yakumo K, Gholami Z, Shaffer D, Stone S, Bayer S, Wray C, Bogden R, Dayananth P, Ward J, Tonin P, Narod S, Bristow P, Norris F, Helverling L, Morrison P, Rosteck P, Lai M, Barrett J, Lewis C, Neuhausen S, Cannon-Albright L, Goldgar D, Wiseman R, Kamb A, Skolnick M (1994) A strong candidate for the breast and ovarian cancer susceptibility gene BRCA1. Science 266: 66–71 7545954 10.1126/science.7545954

[bib26] Moller P, Borg A, Evans D, Haites N, Reis MM, Vasen H, Anderson E, Steel CM, Apold J, Goudie D, Howell A, Lalloo F, Maehle L, Gregory H, Heimdal K (2002) Survival in prospectively ascertained familial breast cancer: Analysis of a series stratified by tumour characteristics, BRCA mutations and oophorectomy. Int J Cancer 101: 555–559 12237897 10.1002/ijc.10641

[bib38] Morrison PJ, Steel CM, Vasen HFA, Eccles D, Evans DGR, Moller P, Hodgson S, StoppaLyonnet D, ChangClaude J, Caligo M, Olah E, Haites NE, Nevin NC (1999) Insurance implications for individuals with a high risk of breast and ovarian cancer in Europe. Disease Mark 15(1–3): 159–165 10.1155/1999/748254 PMC3851117 10595272

[bib39] Morrison PJ, Steel CM, Nevin NC, Evans DG, Eccles D, Vasen H, Moller P, Hodgson S, Stoppa-Lyonnet D, Chang-Claude J, Caligo M, Olah E, Haites NE (2000) Insurance considerations for individuals with a high risk of breast cancer in Europe: some recommendations. CME J Gynecol Oncol 5: 272–277

[bib27] Morrison P (2001) Insurance, genetic testing and familial cancer: recent policy changes in the United Kingdom. Ulster Med J 70: 79–88 11795771 PMC2449245

[bib28] Rebbeck TR, Lynch HT, Neuhausen SL, Narod SA, Van't Veer L, Garber JE, Evans G, Isaacs C, Daly MB, Matloff E, Olopade OI, Weber BL, The Prevention Observation of Surgical End Points Study Group (2002) Prophylactic oophorectomy in carriers of BRCA1 or BRCA2 mutations. N Engl J Med 346: 1616–1622 12023993 10.1056/NEJMoa012158

[bib29] Reichelt J, Dahl A, Heimdal K, Moller P (1999) Uptake of genetic testing and pre-test levels of mental distress in Norwegian families with known BRCA1 mutations. Dis Markers 15: 139–143 10595268 10.1155/1999/581346 PMC3850804

[bib30] Schwartz MD, Peshkin BN, Hughes C, Main D, Isaacs C, Lerman C (2002) Impact of BRCA1/BRCA2 mutation testing on psychologic distress in a clinic-based sample. J Clin Oncol 20: 514–520 11786581 10.1200/JCO.2002.20.2.514

[bib31] Smith K, West J, Croyle R, Botkin J (1999) Familial context of genetic testing for cancer susceptibility: moderating effect of siblings' test results on psychological distress one to two weeks after BRCA1 mutation testing. Cancer Epidemiol Biomarkers Prevent 8: 385–392 10207644

[bib32] Stoutjesdijk MJ, Boetes C, Jager GJ, Beex L, Bult P, Hendriks J, Laheij R, Massuger L, van Die L, Wobbes T, Barentsz J (2001) Magnetic resonance imaging and mammography in women with a hereditary risk of breast cancer. J Natl Cancer Inst 93: 1095–1102 11459871 10.1093/jnci/93.14.1095

[bib33] Warren RML, Pointon L, Caines R, Hayes C, Thompson D, Leach MO, The UK MRI Breast Screening Study (MARIBS) (2002) What is the recall rate of breast MRI when used for screening asymptomatic women at high risk? Magnet Reson Imag 20: 557–656 10.1016/s0730-725x(02)00535-0 12413602

[bib34] Watson M, Lloyd S, Davidson J, Meyer L, Eeles R, Ebbs S, Murday V (1999) The impact of genetic counselling on risk perception and mental health in women with a family history of breast cancer. Br J Cancer 79: 868–874 10070883 10.1038/sj.bjc.6690139 PMC2362694

[bib35] Wooster R, Bignell G, Lancaster J, Swift S, Seal S, Mangion J, Collins N, Gregory S, Gumbs C, Micklem G (1995) Identification of the breast susceptibility gene BRCA2. Nature 378: 789–791 8524414 10.1038/378789a0

